# Supplementary material for: Hypervirulent Klebsiella pneumoniae causing bloodstream infections in Hungary
Source: Microbiol Spectr. 2025 Aug 4;13(9):e00031-25. doi: 10.1128/spectrum.00031-25 (PMC12403626; doi:10.1128/spectrum.00031-25)
Supplement: Supplemental figure and tables — Fig. S1 and Tables S1 to S7. [file spectrum.00031-25-s0001.pdf]

Supplementary Figure 1. Virulence plasmids' Mash distance heatmap

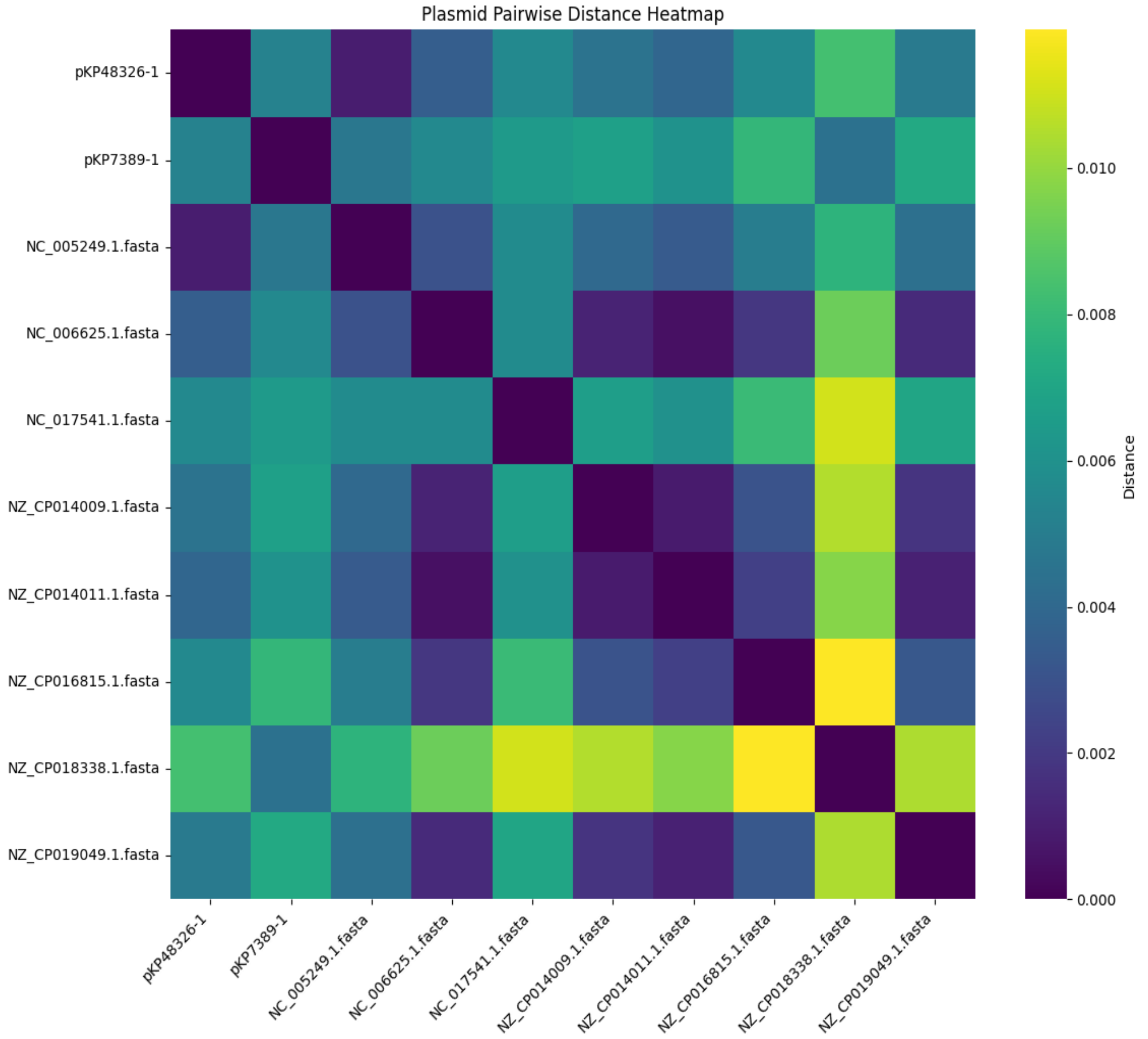

Distances are marked by color with the color scale shown on the figure

Supplementary Table 1. Characteristics of *Klebsiella pneumoniae* ST86, ST5 and ST893 isolates from the BigSdb database to which the Hungarian isolates were compared to

ID	Isolate	Isolation year	country	source type	Sequence type
325	CIP 52.204	0	unknown	Unknown	ST86
1511	SA1	2008	France	Human- blood	ST86
1513	100519185	2010	France	Human- blood	ST86
1514	hvKP1	0	USA	Human- blood(Liver abscess)	ST86
1528	T88	2009	France	Human- Cerebrospinal fluid	ST86
1530	CG43	0	Taiwan	Human- Liver abscess	ST86
1532	IPEUC-340	1975	France	Human- Septicemia and meningitis	ST86
1722	CG43	0	Taiwan	Human -liver pus- liver abscess	ST86
1754	UCICRE 6	0	source lab USA	Unknown	ST86
2731	SB4936	2014	Guadeloupe	Human- CSF- meningitis	ST86
9637	BJKP01	2015	France	Human- CSF- meningitis	ST86
12713	SB6612	2019	France	Human	ST86
12978	SPARK_1213_C1	2018	Italy	Human-Blood	ST86
13811	SPARK_2609_C1	2018	Italy	Human-faeces	ST86
14338	SPARK_802_C1	2017	Italy	Human-sputum	ST86
15877	PP9	2017	Madagascar	Human -sputum- colonisation	ST86
15879	PP12	2017	Madagascar	Human -sputum- colonisation	ST86
24223	ERR4463246	0	Germany	Human	ST86

24955	DRR280986	2017	Japan	Human	ST86
24965	DRR280997	2017	Japan	Human	ST86
24967	DRR280999	2017	Japan	Human	ST86
24968	DRR281000	2017	Japan	Human	ST86
24976	DRR281008	2018	Japan	Human	ST86
24985	DRR281017	2018	Japan	Human	ST86
24986	DRR281018	2018	Japan	Human	ST86
24987	DRR281019	2018	Japan	Human	ST86
24998	DRR281030	2018	Japan	Human	ST86
25003	DRR281036	2018	Japan	Human	ST86
25004	DRR281038	2018	Japan	Human	ST86
25049	DRR281095	2019	Japan	Human	ST86
25050	DRR281097	2019	Japan	Human	ST86
25062	DRR281111	2019	Japan	Human	ST86
25086	DRR320029	2016	Japan	Human	ST86
25119	DRR320094	2017	Japan	Human	ST86
25124	DRR320102	2017	Japan	Human	ST86
25125	DRR320103	2017	Japan	Human	ST86
25126	DRR320106	2017	Japan	Human	ST86
25127	DRR320107	2017	Japan	Human	ST86
25129	DRR320110	2017	Japan	Human	ST86
25137	DRR320122	2017	Japan	Human	ST86
25164	DRR320160	2015	Japan	Human	ST86
25170	DRR320171	2015	Japan	Human	ST86
25171	DRR320177	2015	Japan	Human	ST86
25173	DRR320180	2015	Japan	Human	ST86
25450	ERR4783064	0	Nigeria	Human-Blood	ST86
25608	ERR4783437	0	Nigeria	Human-Blood	ST86

26778	ERR3162553	2017	India	Human-Blood	ST86
27464	ERR3589645	2017	France	Human-stool	ST86
27804	ERR2586382	2014	Vietnam	Human-blood	ST86
27851	ERR3162579	2017	India	Human-blood	ST86
29616	ERR6293500	2015	Norway	Human-blood	ST86
29999	ERR6384993	2018	South Africa	Human-clinical material	ST86
30052	SRR10028187	2017	Switzerland	Human-blood	ST86
30660	SRR12464969	0	USA	Human	ST86
31462	SRR12783054	2016	Russia	Human-bronchial lavage	ST86
31477	SRR12783036	2014	Russia	Human-throat	ST86
31478	SRR12783034	2014	Russia	Human-throat	ST86
31480	SRR12783031	2014	Russia	Human-throat	ST86
32270	ERR5903323	2014	Lithuania	Human Sample	ST86
33135	SRR10905731	2015	USA	Lung	ST86
33682	ERR926757	2014	Guadeloupe	CSF	ST86
33710	SRR12233458	0	Vietnam	Nasal swab	ST86
33734	SRR10160925	2015	Singapore	Stool/rectal swab	ST86
33737	SRR12233393	0	Vietnam	Broncho-alveolar lavage	ST86
33749	SRR12233465	0	Vietnam	Nasal swab	ST86
33775	SRR10160926	2014	Singapore	Rectal	ST86
33968	SRR12233562	0	Vietnam	Broncho-alveolar lavage	ST86
34091	SRR12233400	0	Vietnam	Broncho-alveolar lavage	ST86
34230	SRR12233543	0	Vietnam	Nasal swab	ST86
34317	SRR12233394	0	Vietnam	Broncho-alveolar lavage	ST86
34347	SRR12233397	0	Vietnam	Broncho-alveolar lavage	ST86
34427	SRR12233395	0	Vietnam	Broncho-alveolar lavage	ST86
34472	SRR12233542	0	Vietnam	Nasal swab	ST86

34542	SRR12233470	0	Vietnam	Nasal swab	ST86
34552	SRR12233401	0	Vietnam	Broncho-alveolar lavage	ST86
34553	SRR12233565	0	Vietnam	Broncho-alveolar lavage	ST86
34568	SRR12233560	0	Vietnam	Broncho-alveolar lavage	ST86
34615	SRR12233545	0	Vietnam	Nasal swab	ST86
34633	SRR12233396	0	Vietnam	Broncho-alveolar lavage	ST86
34697	SRR12233457	0	Vietnam	Nasal swab	ST86
35645	SRR14449546	2018	Singapore	urine	ST86
36986	SRR15340826	2020	China	urine	ST86
37065	SRR15858621	2017	China	sputum	ST86
38089	SRR15695926	0	Singapore	Unknown	ST86
38090	SRR15695930	0	Singapore	Unknown	ST86
38095	SRR15695947	0	Singapore	Unknown	ST86
38216	SRR16079165	0	Singapore	Unknown	ST86
38217	SRR16079169	0	Singapore	human	ST86
38580	SRR16761279	0	UK	Unknown	ST86
39990	SRR17640812	2019	India	urine	ST86
40063	SRR17799537	2021	Italy	Broncho-alveolar lavage	ST86
40853	SRR9696996	2011	Australia	clinical sample	ST86
40860	SRR9733707	2007	unknown	Unknown	ST86
40861	SRR9733706	2007	unknown	Unknown	ST86
40865	SRR9733702	2007	unknown	Unknown	ST86
41283	SRR18207174	2002	Australia	wound	ST86
41441	SRR18208918	2020	Australia	swab	ST86
41450	SRR18208932	2020	Australia	Donor Site	ST86
41490	SRR18208991	2020	Australia	urine	ST86
41597	SRR18209141	2020	Australia	Urine - MSU	ST86
42558	SRR6474858	2015	Vietnam	Tracheal fluid	ST86

42745	SRR6474855	2015	Vietnam	Nasopharynx	ST86
43015	SRR6474856	2015	Vietnam	Blood	ST86
43241	SRR6474862	2015	Vietnam	Tracheal fluid	ST86
43339	SRR3051156	2008	UK	Blood	ST86
43661	SRR7657836	2013	New Zealand	Wild animal-Joint swab	ST86
43782	SRR6474860	2015	Vietnam	Tracheal fluid	ST86
43808	SRR7657833	2012	New Zealand	Wild swab- joint swab	ST86
44378	SRR5082332	2013	Australia	Blood	ST86
44501	SRR6474863	2015	Vietnam	Pus	ST86
44644	SRR6474857	2015	Vietnam	Blood	ST86
44659	SRR5973363	2017	USA	Urine	ST86
44771	SRR9217891	2014	Singapore	Rectal swab	ST86
44953	SRR9616070	2015	France	Urine	ST86
44955	SRR4302296	2014	Singapore	Human-Unknown	ST86
45171	SRR5082333	2009	Australia	Blood	ST86
45219	SRR5082343	2008	Australia	Blood	ST86
45288	SRR5082334	2008	Australia	Blood	ST86
45513	SRR6474853	2015	Vietnam	Blood	ST86
45558	SRR6474854	2015	Vietnam	Nasopharynx	ST86
45638	SRR6474864	2015	Vietnam	Blood	ST86
45767	SRR6474861	2015	Vietnam	Tracheal fluid	ST86
45920	SRR6474859	2015	Vietnam	Tracheal fluid	ST86
46147	SRR7657834	2003	New Zealand	wild animal-Brain	ST86
46493	SRR6852549	2017	China	Human-Unknown	ST86
46535	SRR6852550	2017	China	Human-Unknown	ST86
46795	GCA_900079675.1	0	Mexico	Unknown	ST86
46888	GCA_004332075.1	0	Canada	Unknown	ST86
47003	GCA_900493665.1	0	Thailand	Unknown	ST86

47039	GCA_900494005.1	0	Thailand	Unknown	ST86
47577	GCA_003573715.1	0	New Zealand	Unknown	ST86
47578	GCA_003571125.1	0	New Zealand	Unknown	ST86
47735	GCA_000474015.1	0	unknown	Unknown	ST86
48261	GCA_003571145.1	0	New Zealand	Unknown	ST86
48295	GCA_003573725.1	0	New Zealand	Unknown	ST86
48297	GCA_003573775.1	0	New Zealand	Unknown	ST86
48298	GCA_003573755.1	0	New Zealand	Unknown	ST86
48733	GCA_900501255.1	0	Greece	Unknown	ST86
49215	GCA_900515755.1	0	Italy	Unknown	ST86
49287	GCA_900508485.1	0	Lithuania	Unknown	ST86
49309	GCA_900515685.1	0	Latvia	Unknown	ST86
49558	GCA_900505695.1	0	Romania	Unknown	ST86
49650	GCA_900507145.1	0	Serbia	Unknown	ST86
50045	GCA_000813205.1	0	unknown	Unknown	ST86
50055	GCA_000338255.1	0	USA	Unknown	ST86
50056	GCA_003856355.1	0	USA	Unknown	ST86
50622	GCA_002116885.1	0	USA	Unknown	ST86
51111	GCA_900451675.1	0	unknown	Unknown	ST86
51140	GCA_002944845.1	0	China	Unknown	ST86
51332	GCA_003194935.1	0	unknown	Unknown	ST86
51339	GCA_003195035.1	0	unknown	Unknown	ST86
51344	GCA_003195015.1	0	unknown	Unknown	ST86
51345	GCA_003195115.1	0	unknown	Unknown	ST86
51964	GCA_000529645.1	0	unknown	Unknown	ST86
52125	GCA_004311305.1	0	Japan	Unknown	ST86
52169	GCA_004312645.1	0	Japan	Unknown	ST86
52181	GCA_004313025.1	0	Japan	Unknown	ST86

52204	GCA_004313585.1	0	Japan	Unknown	ST86
52292	GCA_000566765.1	0	USA	Unknown	ST86
52321	GCA_000492555.1	0	unknown	Unknown	ST86
53709	GCA_003571155.1	0	New Zealand	Unknown	ST86
53710	GCA_003571075.1	0	New Zealand	Unknown	ST86
54001	GCA_003571065.1	0	New Zealand	Unknown	ST86
29609	ERR6293485	2015	Norway	Human-blood	ST893
34483	SRR10160928	2014	Singapore	Human-rectal swab	ST893
36455	SRR14537207	2016	USA	Human- respiratory	ST893
36458	SRR14537204	2017	USA	Human- respiratory	ST893
37718	SRR13350376	2019	Belgium	Human -tissue sample	ST893
37719	SRR13350377	2016	Belgium	Human -tissue sample	ST893
37720	SRR13350378	2016	Belgium	Human -tissue sample	ST893
37818	SRR15348078	2019	Australia	farm animal-tracheal aspirate	ST893
37820	SRR15348080	2019	Australia	farm animal-tracheal aspirate	ST893
73990	SRR15695624	0	Singapore	Unknown	ST893
43620	SRR9217871	2014	Singapore	Human-rectal swab	ST893
12687	B18168	2018	Ireland	Enviroment sewage	ST5
13843	SPARK_2668_C1	2018	Italy	Dog rectal swab	ST5
27130	ERR1308093	2003	UK	Wild animal tonsil	ST5
27371	ERR486387	2008	UK	Human blood	ST5
27773	ERR2272686	2009	Spain	Human unknown	ST5
28647	ERR2272685	2011	Spain	Human unknown	ST5
48923	GCA_900506315.1	0	Ireland	unknown	ST5
48925	GCA_900506325.1	0	Ireland	unknown	ST5

49665	GCA_900503005.1	0	Slovenia	unknown	ST5
53772	GCA_900085255.1	0	UK	unknown	ST5

Supplementary Table 2. Short read statistics (FastQC)

ID	Number of reads	Mean read length (bp)	GC content (%)	Q20 (%)	Q30 (%)
873_R1	35,708,355	150.3	56	99.99	98.21
873_R2	35,708,355	150.7	56	99.29	95.13
7389_R1	7,963,609	150.5	57	99.98	98.99
7389_R2	7,963,609	150.7	57	99.82	96.48
48326_R1	9,607,671	150.5	57	99.99	98.95
48326_R2	9,607,671	150.4	57	99.83	97.37

Supplementary Table 3. Long read statistics (LongReadSum)

ID	Total reads	Total bases	Longest read length (bp)	Mean read length (bp)	N50 (bp)	GC content (%)	Q10 (%)	Q20 (%)
873_PacBio	245,845	2,526,310,032	47,327	10,276	11,665	57.2	NA	82.24
7389_NanoPore	9,211	94,230,346	147,515	10,230	20,286	56.3	47.71	NA
48326_NanoPore	10,637	143,312,468	154,026	13,473	24,456	56.2	48.45	NA

NA=not analyzed

Supplementary Table 4. Assembly statistics (QUAST)

ID	Number of contigs	Largest contig (bp)	Total length (bp)	N50 (bp)	L50	GC content (%)
873_Unicycler	3	5,320,900	5,553,410	5,320,900	1	57.01
7389_Unicycler	3	5,244,428	5,568,047	5,244,428	1	57.11
48326_Unicycler	2	5,187,926	5,414,661	5,187,926	1	57.32

Supplementary Table 5. Mapping of short reads to contigs (Samtools)					
ID	Contig	Coverage	Mean depth	Mean baseQ	Mean mapQ
873_Unicycler	1	100	1871.1	36	40.2
	2	100	972.02	35.9	39.6
	3	100	3513.65	35.8	40.6
7389_Unicycler	1	100	422.403	36	40.5
	2	100	244.783	35.9	40
	3	100	190.167	35.9	40.9
48326_Unicycler	1	100	496.025	36.2	40.3
	2	100	408.457	36.2	40.1

Supplementary Table 6. Taxonomic composition and contamination of raw reads (Kraken2-Bracken)			
ID	Most abundant taxa (top 3)		
873 short reads	98.77% *Klebsiella pneumoniae*	0.44% *Escherichia coli*	0.39% *Citrobacter portucalensis*
873 long reads	99.91% *Klebsiella pneumoniae*	0.09% *Citrobacter portucalensis*	
7389 short reads	99.70% *Klebsiella pneumoniae*	0.09% *Klebsiella variicola*	0.06% *Klebsiella quasipneumoniae*
7389 long reads	100.00% *Klebsiella pneumoniae*		
48326 short reads	99.73% *Klebsiella pneumoniae*	0.08% Klebsiella variicola	0.06% *Klebsiella quasipneumoniae*
48326 long reads	100.00% *Klebsiella pneumoniae*		

Supplementary Table 7. Contamination in assembled contigs (CheckM)				
ID	Marker lineage	Completeness (%)	Contamination (%)	Strain heterogeneity (%)
873_Unicycler	f__Enterobacteriaceae (UID5121)	100.00	0.11	0.00
7389_Unicycler	f__Enterobacteriaceae (UID5121)	100.00	1.20	0.00
48326_Unicycler	f__Enterobacteriaceae (UID5121)	100.00	0.73	0.00
